# Deciphering Spectroscopic Signatures of Competing Ca^2+^ - Peptide Interactions

**DOI:** 10.1021/acs.jpcb.4c04760

**Published:** 2024-10-22

**Authors:** Carola S. Krevert, Lucas Gunkel, Johannes Sutter, Raphael Meyer, Paul Schneider, Yuki Nagata, Johannes Hunger

**Affiliations:** † Department of Molecular Spectroscopy, Max Planck Insitute for Polymer Research, Ackermannweg 10, Mainz 55128, Germany; ‡ Department of the Synthesis of Macromolecule, Max Planck Insitute for Polymer Research, Ackermannweg 10, Mainz 55128, Germany

## Abstract

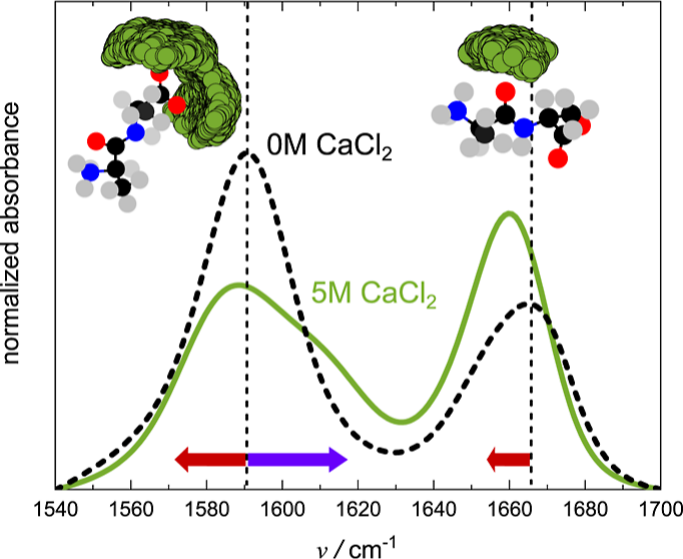

Calcium-protein interactions are of paramount importance in biochemistry. They are a key element in a number of biological processes, such as neuronal signaling. Therefore, an understanding of the interaction at the molecular level is highly desirable. Here, we study the zwitterionic model peptide l-alanyl-*l*-alanine (2Ala), which has two distinct and competing binding sites for Ca^2+^: The carbonyl of the peptide bond and the *C*-terminus, the carboxylate group. We perform linear and two-dimensional IR spectroscopy experiments and find that the spectroscopic signatures of both moieties in the IR spectra change in amplitude and peak position upon the addition of CaCl_2_: A blueshift of the asymmetric carboxylate band and a redshift for the amide I mode. Ab initio molecular dynamics simulations confirm the direct interaction of the Ca^2+^ ion at both the carboxylate and the amide CO site leading to different spectral responses. The blueshift of the asymmetric carboxylate band is caused by a localization of the charge, leading to a decoupling of the CO stretching modes of the carboxylate group. The slight redshift of the amide I mode of 2Ala upon the addition of CaCl_2_ contrasts the blueshift that has been observed for isolated amide motifs, such as *N*-methylacetamide (NMA). This difference is caused by the smaller number of water molecules being replaced by the Ca^2+^ ion for 2Ala’s amide compared to less sterically hindered, isolated amide carbonyls, in conjunction with vibrational Stark effects. Our results highlight the importance of considering potential competing binding sites, such as the amide CO backbone, the termini and residues, as well as the nature of the hydration of both peptide and ion, when exploring ions’ interacting with small peptides and larger proteins.

## Introduction

Since the seminal work by Hofmeister in 1888^1^ the interaction of ions with peptides and proteins in solution has been intensively studied.^2−18^ Among cations, bivalent cations have been shown to markedly interact with proteins and peptides.^12^ Protein-cation interactions are utilized by nature in a plethora of biochemical processes, such as in signaling by ions or metal ions as enzymatic cofactors.^19−21^ In particular, the ubiquitous divalent cation Ca^2+^ plays an important role in many biologically relevant processes.^22^ Calcium ions bind to proteins, causing them to change in their three-dimensional structure. This alteration in protein conformation indirectly regulates cellular processes by affecting protein function, which is crucial for signaling pathways.^22,23^

To understand the effect of Ca^2+^ on the conformation of proteins,^23^ it is quintessential to understand the interaction of Ca^2+^ with proteins on a molecular level. However, the molecular details of such ion–protein interactions in aqueous environments are highly complex. These interactions involve pairwise ion–peptide interactions and competing interaction with the solvent water (i.e., the hydration of the ion and the peptide).^24^

Vibrational spectroscopies have offered valuable insights into ion–protein interactions at elevated salt concentrations since the amide I (CO) vibration of the peptide/protein backbone has been shown to sensitively report on variations in its direct vicinity, such as binding of ions.^11,13,18,25,26^ The vibrational response of amides or ketones in the presence of salts has indicated negligible^12,27^ or very moderate^13,28,29^ effects of monovalent anions and cations on the CO moiety. Similarly, the CO groups of lipids have been shown to be little affected by salts, even for bivalent cations.^19,20^ These minimal ion-specific effects have been associated with weak interaction of these ions with the biomolecules.^12^ Conversely, bivalent cations have been demonstrated to markedly shift the CO vibration of isolated CO groups.^11,12,17,18,29^ Although these shifts are most pronounced at high salt concentrations—much higher than physiological concentrations—weak binding has also been inferred at low concentrations.^12^ These spectral shifts in the presence of cations with a high surface charge density have been ascribed to a salt-induced variation of the hydration of the CO groups,^11,17^ contact ion-pair formation,^12^ or to solvochromatic shifts due to the electric field of the ions exerted on the CO moiety.^29^ Interestingly, in contrast to isolated amide moieties, the amide group of short model peptides with adjacent *N*- and *C*-termini is rather insensitive to the addition of salts.^13,30^ For instance, experiments studying the effect of CaCl_2_ on a tripeptide have suggested that one of the two peptide’s amide vibrations undergoes a minor redshift while for the other amide vibration, a weak, blueshifted shoulder - similar to the findings for isolated amides - appears.^30^ Rather, the carboxylate vibrations of the peptides’ *C*-terminus^13,30^ have been shown to be largely affected by the addition of salts, which can be rationalized by the marked interaction of carboxylates with cations with a high surface charge density.^31,32^ The salt-induced spectral response of the carboxylate resembles findings for oxalate, for which a blueshifted shoulder of the asymmetric stretching vibration is observed upon the addition of salt.^33^

However, the molecular-level origins of the markedly different response of the CO group for isolated CO moieties and peptides have remained elusive. Based on the knowledge for isolated CO groups, the insensitivity of peptides’ amide bonds to salts may stem from weaker interaction of ions with the amide CO due to competing interaction sites at the charged termini,^13^ differences in the local electric fields experienced by the CO due to the electric fields of the zwitterionic peptide,^29^ or due to different hydration of the amide CO in peptides as compared to isolated amide groups.^11^

To elucidate the different behavior of amide groups for isolated amides and peptides, we investigate the effect of CaCl_2_ (0–5 M) on the zwitterionic model peptide l-alanyl-*l*-alanine (2Ala) using infrared (IR) spectroscopy by studying the amide I (CO) vibration and the asymmetric carboxylate stretching vibration of 2Ala. With increasing CaCl_2_ concentration, we find marked spectral changes to the carboxylate mode with a minor redshift at low salt concentrations and the emergence of a blueshifted carboxylate shoulder at high Ca^2+^ concentrations. These spectral changes are also reflected in the spectral dynamics observed by two-dimensional (2D) IR spectroscopy: In the presence of calcium, the carboxylate band becomes more heterogeneous. Conversely, the effect of CaCl_2_ on the amide I band is less pronounced: We find a moderate redshift of the amide I band, which contrasts the blueshifted vibration observed for isolated amide moieties.^11,12,17^ 2D IR experiments confirm weak, yet detectable effects of CaCl_2_ on the vibrational dynamics of the amide CO, predominantly reflected in the vibrational lifetimes. To relate the observed spectral changes to the interaction of ions with 2Ala, we perform ab initio molecular dynamics (MD) simulations combined with single point density functional theory (DFT) calculations. The simulations provide evidence for the direct interaction of Ca^2+^ with both, the CO and COO^–^ groups of 2Ala, yet with a higher probability for Ca^2+^—carboxylate than for Ca^2+^—amide CO interactions. MD simulations show that one or two Ca^2+^ ions bind to the carboxylate, which gives rise to a successive blueshift of the asymmetric carboxylate mode due to Ca^2+^ lifting the degeneracy of the CO groups of the carboxylate moiety. Our results suggest that the CaCl_2_-induced red-shift of the amide CO, as opposed to the blueshift of isolated amide motifs, can be attributed to less water molecules being replaced by a Ca^2+^ cation upon direct interaction for the peptide as compared to isolated amides, in conjunction with vibrational Stark effects. Our findings highlight the mutual interplay between ion binding at different binding sites of a peptide and the importance of considering these distinct binding sites within a peptide to understand specific ion effects on peptides and proteins.

## Methods

### Sample Preparation

Calcium chloride (CaCl_2_) was purchased from Sigma-Aldrich (anhydrous powder, ≥97%). CaCl_2_ was dried in vacuo at 200 °C for 2 h and stored in an Ar-filled glovebox. *L*-alanyl-*l*-alanine (2Ala), *N*-methylacetamide (NMA), l-alanine (1Ala), and D_2_O were purchased from Sigma-Aldrich and used without further purification. We synthesized isotope-labeled 2Ala using a procedure described in the Supporting Information (Supplementary Figures 1–3). All solutions were prepared in 1 mL volumetric flasks. For all experiments reported in the main manuscript, the concentration of 2Ala was kept constant at 250 mM. Solutions of NMA and 1Ala were prepared at 125 mM. The concentration of CaCl_2_ was increased from 1 to 5 M at increments of 1 M.

For IR experiments, samples were contained between two CaF_2_ windows (2 mm thickness; 2.54 cm diameter) separated by a 25 μm Teflon spacer for solutions of 2Ala (50 μm for solutions of 1Ala and NMA measurements).

### Linear IR Measurements

Linear infrared absorption spectra were recorded using a Bruker Vertex 70 spectrometer in transmission geometry at frequencies ranging from 400 to 4000 cm^–1^ with a resolution of 4 cm^–1^. The sample compartment of the spectrometer was continuously purged with purified, dry air. Linear IR absorption spectra were reordered prior to the 2D IR experiments.

### 2D IR Measurements

800 nm pulses (35 fs pulse duration, 1 kHz repetition rate, 7 mJ pulse energy) from a regenerative amplified laser system (Coherent, Astrella) were used to pump an optical parametric amplifier (Topas Prime, Coherent) with an NDFG (noncollinear difference frequency generation) stage to generate IR pulses (6 μm wavelength, 18 μJ pulse energy, 400 cm^–1^ fwhm, ∼100 fs pulse duration). The IR pulses were guided to a commercial 2D IR spectrometer (2D Quick IR, PhaseTech Inc.).^34,35^ A detailed description of the experimental setup (including the acousto-optic modulator to generate pump pulse pairs), in which the modulation of an infrared probe pulse transmitted through the sample by excitation of the sample is monitored as a function of time, can be found elsewhere.^13,36^

The excitation frequency has been resolved in the time domain using pump pulse pairs delayed by 0 to 2555 fs (35 fs increments, with a rotating frame at 1400 cm^–1^). Frequency-dependent modulation of the probe beam was detected by dispersing the probe beam onto a 128 × 128 mercury cadmium telluride array detector. Prior to Fourier transformation of the time-domain data at each detection frequency, the time-domain data were zero-padded to 128 data points and filtered with a Hamming window. The 2D IR spectra of pure 2Ala in D_2_O have been taken from ref (13) and reevaluated for the present study.

### Ab Initio MD Simulations

We performed Born–Oppenheimer MD simulations using the CP2K code.^37^ We used the revPBE^38^ exchange–correlation functional together with the empirical van der Waals correction scheme using Grimme’s D3(0)^39^ correction. We used the mixed Gaussian and plane wave approach.^40^ Atomic orbitals were described using the DZVP-GTH basis set. Core electrons were described using norm conserving Goedecker-Teter-Hutter pseudopotentials.^41^ The time step was set to 0.8 fs, and all simulations were performed at 350 K in the *NVT* ensemble using a canonical velocity rescaling thermostat.^42^ The simulation boxes contained 60 water, 6 CaCl_2_, and 1 2Ala in a (13.3 Å)^3^ box, which corresponds to a ∼ 5 M CaCl_2_ solution. Nine simulation runs with different initial geometries were recorded. Each simulation run was equilibrated for ∼24 ps and trajectories were recorded every 8 fs for ∼124 ps per simulation run, resulting in a total trajectory of 1.1 ns. Radial distribution functions (RDF) have been calculated using *VMD: Visual molecular dynamics*.^43,44^ The vibrational density of states (VDOS) was calculated based on the CO bond velocities.

### Single Point DFT Calculations

Vibrational frequencies of 2Ala and an acetate ion with different Ca^2+^ binding motifs were calculated using DFT calculations with the ORCA program package (version 5.0.4).^45^ Geometry optimizations were performed at the PBE,^46^ def2-TZVPP^47^ level of theory. The atom-pairwise dispersion correction with Becke-Johnson damping (D3BJ) was applied.^39,48^ Calculations were performed with a conductor-like polarizable continuum model with the dielectric properties of water. CHELPG partial charges were obtained using the Multiwfn package.^49,50^ All single point DFT calculations are shown in the Supporting Information.

## Results and Discussion

### Linear IR Spectroscopic Signatures of 2Ala—Ca^2+^ Interactions

**Figure 1:**
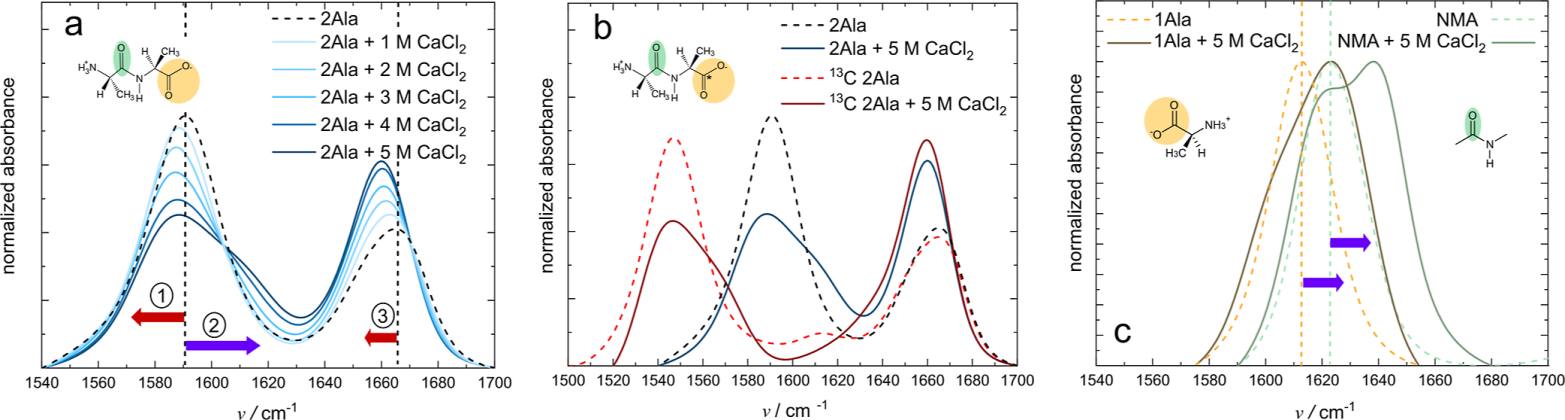
(a) IR spectra of 2Ala in D_2_O at frequencies characteristic for the amide I vibration (∼1660 cm^–1^) and for the asymmetric COO^–^ vibration (∼1590 cm^–1^) with increasing CaCl_2_ concentration. Vertical dashed lines mark the peak positions in the absence of CaCl_2_. Red arrows indicate the salt-induced redshift of the amide I band (③) and the asymmetric COO^–^ (①) at low salt concentrations and the purple arrow the emergence of the blueshifted shoulder of the carboxylate (②) at high salt concentrations. (b) Comparison of the IR spectra of ^13^COO^–^ and ^12^COO^–^ 2Ala with and without 5 M CaCl_2_. All spectral contributions at <1620 cm^–1^ redshift upon isotopic substitution and are therefore assigned to the COO^–^ group. All spectra were corrected for a linear background and normalized to the integrated absorbance (see Supporting Information) (c) linear IR spectra of 1Ala and NMA in D_2_O with and without 5 M CaCl_2_. For 1Ala and NMA addition of CaCl_2_ blueshifts the COO^–^ and the amide I band, respectively.

To explore the effect of calcium on 2Ala, we study the infrared spectra of 2Ala in D_2_O. The IR absorption spectrum of 2Ala (250 mM, black dashed line in Figure 1a) shows the asymmetric stretching vibration of the carboxylate group at 1590 cm^–1^ and the amide I (carbonyl stretching) vibration at 1660 cm^–1^. Upon addition of CaCl_2_, these spectral signatures undergo continuous variation in amplitude and resonance frequency: The carboxylate band decreases in amplitude, redshifts at low salt concentrations (1–3 M) ① and a blueshifted shoulder emerges at ∼1620 cm^–1^ (②) at higher concentrations. The amide I band undergoes a small, yet detectable redshift (by about 7 cm^–1^, ③) and increases in amplitude. These spectral variations are further illustrated by the difference spectra in Supporting Information Figure 4. We note that the spectra in Figure 1a have been corrected for a linear background absorption and normalized to the total peak integral to account for minor variations in the concentrations and optical path lengths, yet the observed spectral changes are also evident from the raw spectra (see Supporting Information Figure 5, see Supporting Information for details on the background correction and normalization).

The spectral variations are similarly present at lower (50 mM) and higher (500 mM) 2Ala concentrations, indicating that self-aggregation of 2Ala does not contribute to the observed spectral changes (see Supporting Information Figure 6). The CaCl_2_ induced spectral changes are also distinctively different from the effect of pH on the 2Ala vibrations (Supporting Information Figure 7), which excludes CaCl_2_-induced changes of 2Ala’s protonation state as cause for the spectral shifts observed in Figure 1a. In contrast to the effect of CaCl_2_ and in line with earlier studies,^12,17^ 2Ala’s infrared spectra are not affected by the addition of NaCl (see Supporting Information Figure 8). As such, our data suggest that the CaCl_2_ induced changes to the amide I and carboxylate vibrations in Figure 1a originate from the interaction of Ca^2+^ with zwitterionic 2Ala.

To elucidate the molecular origin of the Ca^2+^-induced shoulder at 1620 cm^–1^, we performed experiments using ^13^COO^–^ labeled 2Ala. The IR spectra in Figure 1b show that upon isotope-labeling, both the band at 1590 cm^–1^ and the shoulder at 1620 cm^–1^ shift to lower wavenumbers.^51^ As such, the isotope-labeling experiments demonstrate that both the band at 1590 cm^–1^ and the higher frequency shoulder can be assigned to the COO^–^ group, which is further supported by the increase of the shoulder at 1620 cm^–1^ with increasing CaCl_2_ concentration (see fits of the linear IR spectra, Supporting Information Figure 9).

The observed Ca^2+^-induced spectral shifts for 2Ala contrast the effect of CaCl_2_ on simpler molecules. Figure 1c demonstrates that the amide I mode of *N*-methylacetamide and the carboxylate mode of zwitterionic 1Ala both blueshift upon the addition of CaCl_2_. The salt-induced blueshift of the amide I mode confirms earlier studies on NMA^11,17,52^ and is in line with findings for *N*-ethylpropionamide,^18^ and butyramide.^12^

The observed CaCl_2_ induced redshift of the amide I vibration for 2Ala is rather similar to the effect of CaCl_2_ on one of the amide I modes of alanine tripeptide.^30^ Such redshifts of amide I modes have been associated with Stark shifts, originating from the electric field due to cations and anions that accumulate at opposite ends of the CO oscillator.^12,29,53^

As such, our results show that the effect of CaCl_2_ on 2Ala distinctively differs from the salt-induced changes to the amide vibrations of isolated amide groups, suggesting differing interactions. Yet, solely based on the IR absorption spectra it remains open whether the observed spectral changes are due to a gradual variation of the solvent environment (e.g., CaCl_2_ induced dehydration of 2Ala^11,54^) or due to a concentration-dependent variation of distinctively different molecular species with different Ca^2+^ - 2Ala binding geometries.^30,31,55,56^

### 2D IR Spectroscopy of 2Ala and 2Ala—CaCl_2_ Solutions

To spectroscopically decipher different molecular species contributing to the observed vibrational bands, we perform 2D IR experiments. In a 2D IR experiment, a subset of oscillators with a given instantaneous frequency is excited and the sample’s response is probed over a broader detection frequency range. In this manner, the heterogeneity of vibrational bands due to inhomogeneous broadening, such as the distribution of molecular level oscillators with differing resonance frequency due to, e.g., differing environments, can be assessed. Probing the molecular response as a function of time can provide information on the dynamics of these differing molecular-level oscillators.^57^

**Figure 2:**
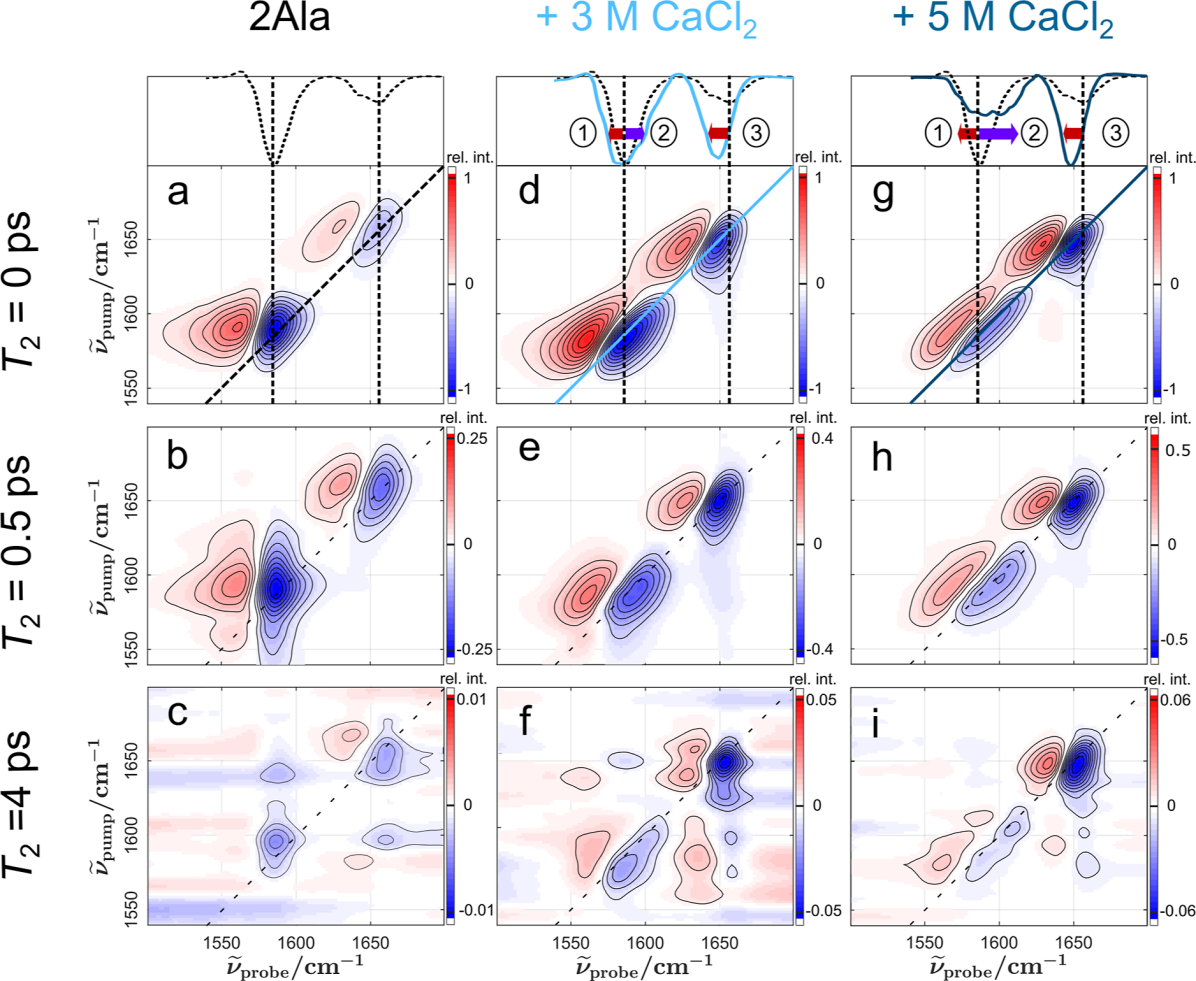
2D IR spectra of 2Ala in D_2_O (a–c) and with 3 M (d–f) and 5 M (g–i) CaCl_2_ added at different waiting times *T*_2_ (top to bottom). Top panels show the diagonal cuts of the 2D IR spectra at *T*_2_ = 0 ps. The dashed line in the top panels corresponds to the diagonal cut in panel a (2Ala in D_2_O). With increasing CaCl_2_ concentration, the carboxylate band becomes more heterogeneous, as evidenced by the elongation of the carboxylate band at all waiting times. This heterogeneity persists up to *T*_2_ = 4 ps (f), (i), at which spectra are dominated by signals due to the heated ground state. Red arrows indicate the salt-induced redshift of the amide I band (③) and the asymmetric COO^–^ (①) at low salt concentrations and the purple arrow the emergence of the blueshifted shoulder of the carboxylate (②) at high salt concentrations.

Figure 2a shows a 2D IR spectrum of 250 mM 2Ala in D_2_O at a waiting time *T*_2_ = 0 ps with ⟨*ZZZZ*⟩ (parallel) polarization combinations. For both vibrational modes, the amide I and the carboxylate band, two signal pairs are detected: A negative signal at the diagonal due to depletion of the vibrational ground state and stimulated emission from the excited state and a red-shifted positive signal due to the anharmonically shifted excited state absorption. The diagonal cut shown in the top panel of Figure 2a resembles the squared linear IR spectrum (see Figure 1).^57^ With increasing time delay between excitation and probe pulse, the signal intensity decreases (Figure 2b, c) due to relaxation to the vibrational ground state. Due to the faster relaxation of the carboxylate mode relative to the amide I mode, the decrease in the signal intensity is more pronounced for the signal pair at 1590 cm^–1^. In addition to the decay of the signal intensity, the shape of the 2D IR signals varies with time: Both signals are elongated along the diagonal at 0 ps waiting time, with the amide I band exhibiting a more pronounced elongation. With increasing *T*_2_ the signals become more vertical, evidencing a loss of correlation of the signal frequency to the excitation frequency. These dynamics are commonly referred to as spectral diffusion. At 4 ps waiting time, where the signals are dominated by thermally induced spectral signatures due to dissipation of the vibrational excess energy (Figure 2c), off-diagonal peaks can be observed.

Addition of 3 and 5 M CaCl_2_ (Figure 2d–i; for other concentrations see Supporting Information Figure 10) to solutions of 2Ala alters these signals: In line with the IR absorption spectra, the relative intensities of the amide I and carboxylate signals are altered. Also the diagonal slices at *T*_2_ = 0 ps resemble the spectra in Figure 1 with an emerging shoulder at 1620 cm^–1^ (②) (Figure 2d, g) and a redshift of the amide I mode (③) with increasing CaCl_2_ concentration.

Remarkably, for high CaCl_2_ concentrations, in particular at 5 M, the loss of frequency–frequency correlations is slowed down (Figure 2) (i): The carboxylate and to a lesser extent the amide I signal are markedly elongated along the diagonal at *T*_2_ = 0.5 and 4 ps, which contrasts the spectra for 2Ala in D_2_O (Figure 2a–c). Our data suggest that these changes to the vibrational structure and dynamics are specific to CaCl_2_, as spectra of 2Ala in the presence of NaCl (Supporting Information Figure 11) resemble the spectra of 2Ala in D_2_O.

### Spectral Heterogeneity and Dynamics from 2D IR

To further quantify the spectral heterogeneity and their dynamics, we evaluate the center line slope (CLS) for both, the amide I and carboxylate signals. We determine the CLS, a measure for the (time-dependent) spectral heterogeneity, from the minima of the bleaching signals parallel to the probe axis. The minima (center line points) are determined by Gaussian fits to the bleaching signals at frequencies for which the initial (*T*_2_ = 0) bleaching signal is less than 10% of the maximum bleaching signal (for details, see Supporting Information Figure 12). The slope of linear fits to the center line points as a function of excitation frequency represents the CLS. Here, a CLS of 1 means a perfect correlation between excitation and detection frequencies, while a CLS value of 0 corresponds to uncorrelated frequencies.

**Figure 3:**
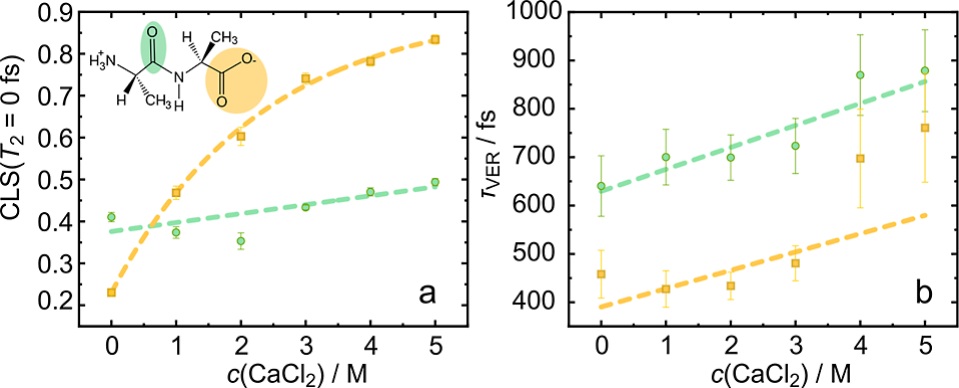
Concentration-dependence of (a) CLS at 0 fs waiting time (instantaneous heterogeneity) and (b) vibrational energy relaxation lifetimes τ_VER_ for the amide I (CO, green) and carboxylate peak (COO^–^, yellow). Dashed lines serve to guide to the eye.

We evaluate the instantaneous heterogeneity by extracting the CLS value at *T*_2_ = 0 fs (see Figure 3a). To determine spectral diffusion dynamics, we fit a monoexponential decay with the rate constant *k*_CLS_ to the time-dependent CLS(*T*_2_) at delay times with sufficiently intense signals (*T*_2_ ≤ 800 fs for the carboxylate and *T*_2_ ≤ 1 ps for the amide I mode, see also discussion of vibrational lifetimes below). The thus obtained *k*_CLS_ values are shown in Supporting Information Figure 12.

When CaCl_2_ is added, we initially observe a decrease in CLS for the amide I mode at *T*_2_ = 0 fs, followed by a subsequent increase of approximately 15%. Yet, the CaCl_2_-induced change is rather moderate and in line with observations for the interaction of 2Ala with monovalent ions (such as LiCl^13^ or NaCl, see Supporting Information Figure 13). As such, CaCl_2_ does not alter the vibrational structure of the amide I mode significantly. However, the CLS(*T*_2_ = 0 fs) for the antisymmetric stretching vibration of the carboxylate mode shows a significant increase by a factor of ∼3.5 (from pure 2Ala to 5 M CaCl_2_ solution). This increase is much more pronounced than for monovalent salts: For LiCl, the instantaneous heterogeneity of the carboxylate mode increases by a factor of ∼2.5,^13^ for NaCl by a factor of 2 (see Supporting Information Figure 13). These findings demonstrate a more pronounced spectral inhomogeneity for the carboxylate band in the presence of CaCl_2_ as compared to NaCl and LiCl, which may point to a broader range of ion–carboxylate interaction motifs or to a stronger interaction and longer-lived binding of CaCl_2_ to the carboxylate: The data suggest the presence of multiple molecular 2Ala-Ca^2+^ (or 2Ala-Cl^–^) species.

To obtain information on the lifetime of the underlying molecular species, one can consider the spectral diffusion dynamics, i.e., the decay rates *k*_CLS_ of the carboxylate peak. We find that the decay rate decreases continuously upon the addition of CaCl_2_ (see Supporting Information Figure 12), and the decay time (1/*k*_CLS_) parallels the variation of CLS(*T*_2_ = 0 fs) with CaCl_2_ concentration. The marked elongation of the signals at *T*_2_ = 4 ps (Figure 2i) demonstrates that, even at waiting times for which the heated ground state dominates the signals (see also discussion of the vibrational relaxation below), the frequency response is still correlated to the excitation frequency, indicating that the underlying molecular-level species are long-lived and spatially separated: At 4 ps the excess energy (heat) is not transferred between initially excited and initially nonexcited carboxylate groups. Interestingly, the slow-down of the spectral dynamics in the presence of CaCl_2_ is comparable to our earlier findings for LiCl.^13^ Conversely, for NaCl only minor changes in the rate constant *k*_CLS_ up to 20% are observed (see Supporting Information Figure 13).

The similarity for LiCl and CaCl_2_ indicates slowed-down spectral diffusion dynamics for both highly viscous ∼5 M salt solutions,^58,59^ yet the altered dynamics do not simply reflect solution viscosity: While the viscosity for aqueous 5 M LiCl solutions increases by a factor of 2 (relative to neat water), the viscosity of a 5 M CaCl_2_ solution increases by a factor of 5. The rate constants of the decay in CLS of the carboxylate mode decreases by a factor of ∼5 for CaCl_2_, while  decreases by a factor of ∼4. Hence, while the decrease in the spectral dynamics can be partly attributed to the increase in viscosity, the dynamics with which the COO^–^ groups loose their frequency memory (i.e., the exchange of their environment) differs from macroscopic dynamics: Exchange of environments around COO^–^ are very similar for LiCl and CaCl_2_, despite their differing viscosities. Again, the slowed down spectral dynamics evidence the presence of different molecular 2Ala-ion species, which barely interconvert on the time scale of a few picoseconds.

### Vibrational Energy Relaxation Dynamics

To further elucidate different molecular-level species, we consider vibrational energy relaxation dynamics—i.e., the decay of the 2D IR signals with time—in more detail. The vibrational energy relaxation lifetime (τ_VER_) of each molecular species is a result of the coupling of the vibrational mode to its microscopic environments and may thus differ for different molecular species, which have different environments.

To explore whether CaCl_2_ affects vibrational energy relaxation (the decay of the vibrationally excited state), we integrate the entire bleaching signals for the amide I and the carboxylate signals (for details on the integration, see Supporting Information Figure 14). The characteristic relaxation time of the integrated signal is determined by fitting a kinetic model^60^ (eq 1) to the evolution of the signal. In this model, the exited state decays with relaxation time τ_VER_ and energy dissipation leads to a persistent modulation due to a heated ground state, with the peak integral of the excited state *V*_exc_ and the peak integral of the heated ground state *V*_heat_
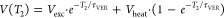
1

This model describes the experimental data very well (see Supporting Information Figure 14) with τ_VER_ ∼ 0.46 ps for the carboxylate mode and τ_VER_ ∼ 0.67 ps for the amide I mode^13^ for 2Ala in D_2_O.^13^ With the addition of CaCl_2_, the vibrational relaxation time increases, for both the amide I and the carboxylate peak (see Figure 3b). Interestingly, although the spectral signatures of the amide I peak are little affected by CaCl_2_, its vibrational energy relaxation dynamics are altered and increase to τ_VER_ = 0.88 ps at 5 M CaCl_2_. For the carboxylate mode, τ_VER_ nearly doubles from 0.46 ps (0 M) to 0.76 ps at 5 M. Conversely, vibrational energy relaxation of both modes for 2Ala is virtually insensitive to the addition of NaCl (see Supporting Information Figure 15). Although the overall relaxation time only provides the average effect of CaCl_2_, this analysis demonstrates that CaCl_2_ alters energy relaxation pathways and the relaxation dynamics have, as such, the potential to discriminate different molecular-level species that contribute to the observed vibrational bands.

**Figure 4:**
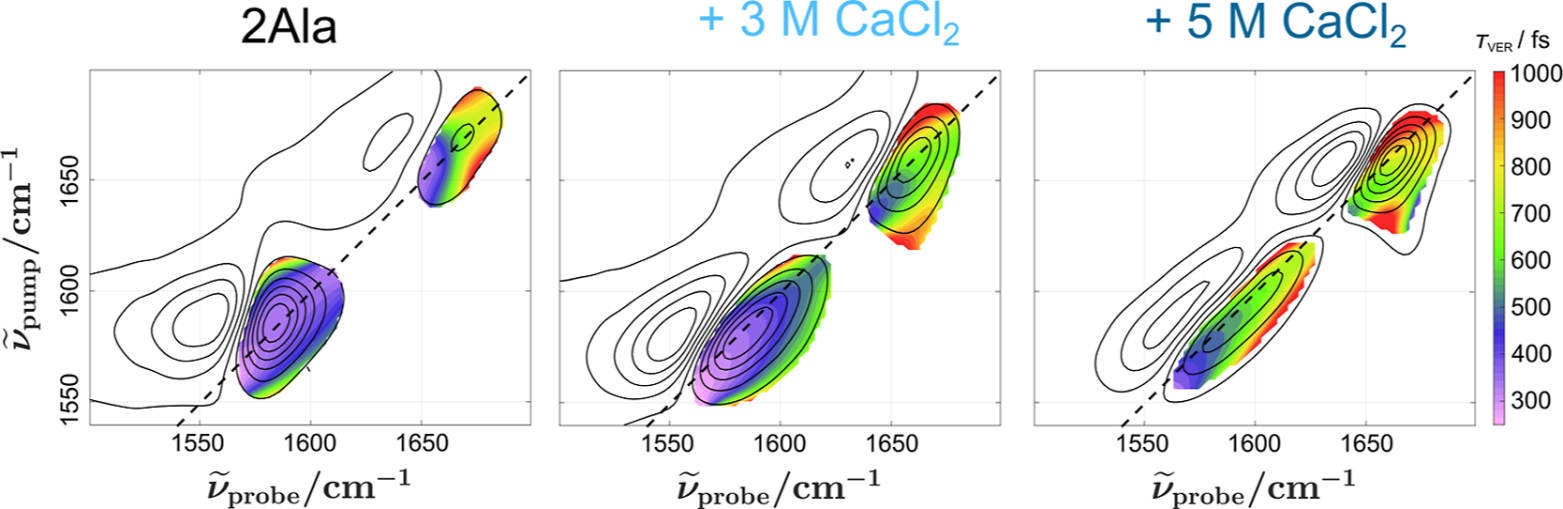
Decay time maps for 2Ala with 0 M(a), 3 M (b), and 5 M (c) CaCl_2_ solutions. For each data point shown here, we integrate a 5 × 5 pixel grid of the 2D IR spectra and fit a monoexponential decay to a heated ground state (see eq 1) to the waiting-time dependent data. The decay time is shown as color map for all regions in the 2D IR spectra (contour lines) with a maximum bleaching signal exceeding 10% of the global maximum the bleaching signal at *T*_2_ = 0 ps.

To elucidate whether distinct molecular species with different vibrational lifetimes underlie the observed changes of the overall τ_VER_ values, we determine decay maps.^25^ This is achieved by integrating the signal of a five-by-five pixel grid of the 2D IR spectra and determining the decay time of the signal analogously to the analysis above using eq 1. The thus obtained decay times are shown for spectral regions where the bleaching signal is smaller than 10% of the maximum bleach at *T*_2_ = 0 ps in Figure 4 for pure 2Ala in D_2_O (a), 3 M CaCl_2_ (b), and 5 M CaCl_2_ (c).

The decay time in these maps at a given spectral position can be affected by both, vibrational energy relaxation and spectral diffusion, and the heterogeneity in the decay times only represents heterogeneous energy relaxation dynamics if spectral diffusion dynamics are distinctively different from the energy relaxation dynamics. For the amide I signal, the time scales for spectral diffusion and energy relaxation are comparable and, as such, the decay time maps exhibit an apparent heterogeneity (Figure 4). Despite the similarity of the relaxation and spectral diffusion time scales, which prevents a more detailed interpretation, this heterogeneity becomes less pronounced with increasing CaCl_2_ concentration, indicative of amide - Ca^2+^ interaction.

Conversely, spectral diffusion and energy relaxation dynamics are distinctively different at low and high CaCl_2_ concentrations for the carboxylate signal. At 0 M CaCl_2_ the decay time map is rather homogeneous, and the observed decay time agrees well with the relaxation time in Figure 3b. At high CaCl_2_ concentration, where the overall vibrational relaxation time is significantly faster than the spectral diffusion dynamics, the decay time maps become heterogeneous: At the red-shifted edge of the carboxylate signal, the decay time resembles the decay time of 2Ala in D_2_O, while at higher frequencies where the shoulder in the linear IR spectra is observed (Figure 1) the decay time is significantly longer. As such, this analysis demonstrates that the observed increase in the overall τ_VER_ values (Figure 3b) is a result of the emergence of molecular species with blueshifted vibrational response and a longer vibrational lifetime in the presence of CaCl_2_.

Again, the emergence of species with distinctively different vibrational lifetimes appears specific to CaCl_2_ as the decay time maps for the carboxylate band in solutions of 2Ala and NaCl (see Supporting Information Figure 15) remain homogeneous even at 5 M NaCl. For decay time maps for 1, 2, and 4 M CaCl_2_-2Ala solutions see Supporting Information Figure 16. Together, the vibrational lifetimes indicate the presence of at least two underlying molecular-level 2Ala species in the presence of CaCl_2_ with different energy relaxation dynamics.

### Ab Initio MD Simulations of 2Ala and 2Ala-CaCl_2_ Solutions

To better understand the molecular-level species that underlie the observed CaCl_2_-induced spectral changes, we performed ab initio MD simulations. As different binding motifs can be readily separated from the trajectories, we limit the simulations to 2Ala in the presence of a high concentration of CaCl_2_ (5 M), at which all relevant CaCl_2_-2Ala species should be transiently formed.

**Figure 5:**
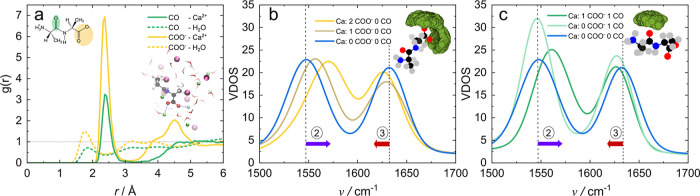
(a) Oxygen-Ca^2+^ (solid lines) and oxygen-H_2_O (dashed lines) RDF for carboxylate (yellow) and amide CO (green), with a simulation snapshot as inset (b) VDOS of 2Ala in 5 M CaCl_2_ with no direct Ca^2+^ interaction (blue), one Ca^2+^ at the carboxylate (dark yellow) and two Ca^2+^ at the carboxylate (yellow). Inset shows the distribution of Ca^2^ + around the carboxylate group of 2Ala. (c) VDOS of 2Ala in 5 M CaCl_2_ solution with no direct Ca^2+^ interaction (blue), direct interaction at the amide CO but not at the carboxylate (bright green), and direct interaction at the carboxylate and the amide CO (dark green). Inset shows the distribution of Ca^2+^ around 2Ala’s amide CO.

We investigate the distribution of Ca^2+^ around 2Ala by calculating the RDF (RDFs) of Ca^2+^ next to 2Ala’s oxygen atoms. The Ca^2+^-O RDFs in Figure 5a, show intense peaks at 2.4 Å for all oxygens of 2Ala (solid lines). We find that this peak is about twice as intense for the carboxylate oxygens (solid yellow line) compared to the amide oxygen (solid green line), indicating a higher density of Ca^2+^ ions adjacent to the carboxylate group than to the amide CO group. The RDF for the carboxylate group (solid yellow line) exhibits a second peak at ∼4.5 Å, which is due to Ca^2+^ ions bound to the other oxygen atom of the carboxylate group, indicative of a monodentate binding being the predominant binding motif. Comparison of the Ca^2+^-2Ala RDFs (solid lines) to the water-2Ala RDFs (dashed lines) shows that the first shell peaks of the Ca^2+^-2Ala RDF are due to direct Ca^2+^-2Ala interactions: The first peak in the carboxylate-water RDF (CO–) is located at 1.78 Å and for the amide oxygen at 1.88 Å. The slight difference in the location of both hydration peaks is also reflected in the oxygen–oxygen RDFs (COO–: 2.83 Å, CO–: 2.98 Å, see Supporting Information Figure 17) and suggests somewhat stronger hydration of the carboxylate group as compared to the amide CO. The location of the first Ca^2+^ peak at shorter distances than the first water oxygen peak confirms that Ca^2+^ directly interacts with the CO oxygen. The RDFs for the chloride ion (see Supporting Information Figure 18) suggest that Cl^–^ directly interacts with both, the amide NH and the *N*-terminus, and the occupancy is correlated to Ca^2+^ interaction with 2Ala. This correlation may enhance vibrational Stark effects upon binding of Ca^2+^ to 2Ala, albeit a high Cl^–^ concentration in NaCl solutions has negligible effect on the vibrational spectra (Supporting Information Figure 8).

To quantify the interplay between the interaction of 2Ala’s oxygens with Ca^2+^ and water, we categorize simulation frames based on the interaction with Ca^2+^ (for more detail, see Supporting Information) and determine the hydration numbers of the functional groups of 2Ala by integrating all water molecules within *d*_CO–H_ ≤ 2.4 Å.

Table 1 indicates that upon direct interaction of Ca^2+^ with the amide CO (1 Ca^2+^ at CO) the amide CO is fully dehydrated with Ca^2+^ replacing all water molecules. The same full dehydration of the carbonyl upon Ca^2+^ interaction has also been reported for NMA.^17^ When Ca^2+^ binds to the oxygen of the carboxylate (1 Ca^2+^ at COO^–^), on average about one water molecule is replaced, yet dehydration due to Ca^2+^ is incomplete. Thus, the analysis of the RDFs illustrates that, despite the very different spectral response observed in the IR experiments, Ca^2+^ ions directly interact with both, the carboxylate oxygens and the amide oxygen of 2Ala. For the amide oxygen, Ca^2+^ ions fully replace hydrating water molecules, while the carboxylate moiety remains partly hydrated.

**Table 1:** Average Number of Water Molecules Present for Categorized Frames with a CO Oxygen—Water Hydrogen Distance **d**_CO–H_ < 2.4 Å as Bonding Criterion for Water

category	average number of water
0 Ca^2+^ at CO	0.73
1 Ca^2+^ at CO	0.01
0 Ca^2+^ at COO^–^	1.64
1 Ca^2+^ at COO^–^	0.38

In addition to the interplay between interaction of water and/or Ca^2+^ with the individual binding sites at 2Ala, we further consider the occurrence of Ca^2+^ at the different binding sites. In addition to simulation frames for which only one Ca^2+^ ion directly binds to only one of 2Ala’s oxygens (26% only at COO^–^; 2% only at CO), we find a significant fraction (26%) of configurations for which two Ca^2+^ ions bind (monodentate) to the two carboxylate oxygens, which is also referred to as a *bridging* binding configuration.^54^ Also configurations with two Ca^2+^ ions binding to one of the carboxylate oxygens and to the amide CO are observed (13%). Yet, we find no evidence for all three oxygen sites (two carboxylate oxygens and one amide oxygen) being simultaneously occupied by Ca^2+^ at the same time or for bidentate coordination of both carboxylate oxygens to Ca^2+^.

To further explore the spectral consequences of the interaction of calcium with 2Ala, we compute the VDOS from the relative CO velocities for different binding states, categorized based on the interaction with Ca^2+^ (see Figure 5b, c). We categorized the frames from the simulation into five categories according to the number of Ca^2+^ bound to each interaction site (see Supporting Information for more details).

Figure 5b illustrates the effect of 0, 1, and 2 Ca^2+^ binding to the carboxylate group on the VDOS, with the site at the amide oxygen not occupied by calcium, while Figure 5c shows the effect of Ca^2+^ binding to the amide CO group. The solid blue lines in Figure 5b and c represent the spectra for species for which 2Ala’s amide I and asymmetric carboxylate vibration do not directly interact with Ca^2+^. The computed spectral shifts upon binding of Ca^2+^ to 2Ala reproduce the experimentally observed trends with increasing CaCl_2_ concentration very well:

Upon binding of one Ca^2+^ ion to the carboxylate, the carboxylate band shifts to higher wavenumbers (dark yellow line in Figure 5b) ②. Such blueshift is commonly observed for calcium binding to one oxygen atom of the carboxylate, with the other oxygen being hydrated by water. This coordination geometry is also often termed *pseudobridging* coordination.^30,31,54,56^ The blueshift of the carboxylate band upon the pseudobridging interaction can be rationalized by the localization of the charge at the interacting oxygen and, consequently, lifting of the degeneracy of the two CO bond potentials (see Supporting Information Figure 19). Single point DFT calculations (see Supporting Information Figure 20) show that the lifted degeneracy causes a spectral blueshift for the CO group of COO^–^ not interacting with Ca^2+^, in contrast to the redshift for a bidentate binding geometry.^31,32,54^

Upon interaction of a second Ca^2+^ with the carboxylate, the VDOS exhibits an additional blueshift (yellow line in Figure 5b). Single point DFT calculations suggest that the asymmetry of the charge distribution at the carboxylate’s oxygens for one and two Ca^2+^ interacting with the carboxylate is rather comparable (Supporting Information Figure 19), and in both configurations the degeneracy of the two CO groups is lifted in a similar manner. The dissimilarity of the two CO groups interacting with two Ca^2+^ ions is further supported by the MD simulations, which show that the oxygen–carbon distances of the two CO groups of COO^–^ differ for a trajectory with bridging Ca^2+^ interaction as opposed to the bond distances in the absence of Ca^2+^ (see Supporting Information Figure 21). The additional blueshift upon interaction with a second calcium may be rationalized in analogy to the interaction of Ca^2+^ with isolated carbonyls: For the interaction of CO with water, electron density is transferred from the carbonyl to the σ* orbital of water, weakening the CO bond and leading to a redshift. When water is replaced by Ca^2+^, the transfer of electron density is no longer possible, leading to a blueshift, depending on the number of water molecules replaced by the ion.^12,17,29,61,62^ Together, the gradual blueshift of the carboxylate band with additional Ca^2+^ ions ② interacting with the carboxylate predicted by the MD simulations is in line with the gradual increase of the heterogeneity of the carboxylate band as concluded from the CLS (Figure 3a): The enhanced heterogeneity of the carboxylate vibration concluded from the 2D-IR spectra can be explained by a blueshift of the vibrational mode due to the successive binding of Ca^2+^ to the two oxygen sites at the carboxylate group with increasing CaCl_2_ concentration.

The simulations at high CaCl_2_ concentrations do not provide insights into the initial redshift of the carboxylate band ① at low salt concentrations. Yet, for amide CO similar redshifts have been reported at low salt concentrations and ascribed to Stark shifts, which may also be the origin of the observed redshift for the carboxylate.^29,53^

The experimentally observed redshift of the amide I mode ③ is also reproduced in the VDOS spectra (see Figure 5c, dark and bright green line). As such, also the simulations confirm that the spectral response of the amide I mode to Ca^2+^ interaction markedly differs from the response of isolated amide groups.^11,12,17,29^ To explore the molecular origins of this different behavior, we disentangle the VDOS of the amide group for three different configurations: (i) One water molecule hydrating the amide oxygen, (ii) one Ca^2+^ ion bound to the amide oxygen, and (iii) neither water nor Ca^2+^ bound to the amide oxygen (see Supporting Information Figure 22). The comparison of these three configurations shows that both, direct interaction between the amide and water (i) and the amide and Ca^2+^ (ii) induce a similar redshift as compared to the configurations with no interaction at the amide (iii). As such, the replacement of water by Ca^2+^ in the vicinity of the amide oxygen does not result in appreciable changes in the instantaneous frequency of the amide I mode: The simulated spectra explain the weak sensitivity of the amide I frequency to the presence of Ca^2+^, as opposed to the response of isolated amide groups.^11,12,17,29^ This insensitivity of the amide I frequency to the molecular identity of its direct environment is in line with the experimentally observed insensitivity of the CLS to addition of CaCl_2_ (Figure 3a). The moderate redshift of the amide I mode with increasing CaCl_2_ concentration (Figure 1a) is therefore likely related to a transition from an incompletely hydrated amide CO group at low concentrations to configurations with Ca^2+^ bound to the amide moiety at high CaCl_2_ concentrations. Despite the insensitivity of the amide I mode frequency to Ca^2+^ interaction, the presence of a water molecule or a Ca^2+^ ion in the vicinity of the amide oxygen will most likely alter the density of states with lower energy and coupling of the amide I mode to those states, which is intimately connected to vibrational energy relaxation of the amide I mode. Therefore, the replacement of water molecules in the vicinity of the amide group by Ca^2+^ ions can explain the observed variation of the vibrational energy relaxation dynamics upon addition of CaCl_2_ (Figures 3b and 4).

The markedly different spectral response of more isolate amide groups to CaCl_2_ (see Figure 1c and refs (11, 12, 17 and 29)) can in turn be rationalized by the different hydration states of isolated amide groups and the sterically less accessible amide moiety of 2Ala: More sterically accessible amide groups like in NMA are hydrated by about two water molecules, which are nearly fully replaced by one Ca^2+^ ion^17^ upon addition of CaCl_2_. This replacement leads to a blueshift due to a reduction of electron transfer from the environment to the σ* orbitals for Ca^2+^ bound to the amide as compared to the amide hydrated by two water molecules.^12,61^ Our results suggest that the spectral consequences for the sterically less accessible amide CO of 2Ala are similar since less than one hydrating water molecule is replaced by Ca^2+^. Hence, the accessibility to hydration water of the amide group seems to be pivotal for the sensitivity to interaction with Ca^2+^. Such steric arguments should also apply to amide groups in a protein backbone in general, suggesting that the amide I mode frequency is not an ideal probe for detecting amide-Ca^2+^ interactions. However, the altered energy relaxation pathways offer the potential to spectroscopically detect amide-Ca^2+^ interaction via the vibrational energy relaxation time scale.

## Conclusion

Herein, we examined the interaction between the zwitterionic dipeptide l-alanyl-*l*-alanine (2Ala) and the physiologically relevant, strongly hydrated bivalent salt CaCl_2_. Two distinct interaction sites at 2Ala have been examined with IR spectroscopy: The backbone CO (amide I mode) and *C*-terminus (asymmetric carboxylate mode). Linear IR spectra of 2Ala with increasing concentration of CaCl_2_ show changes in peak position and amplitude for both modes and can be summarized in the following three major trends: ① The asymmetric carboxylate stretching peak initially experiences a redshift, followed by ② a blueshift at concentrations above 2 M CaCl_2_. ③ The amide I mode, on the other hand, experiences a redshift with increasing salt concentration. Model systems that only contain either the amide I mode (NMA) or the carboxylate mode (zwitterionic alanine), both show a blueshift upon adding CaCl_2_. 2D IR experiments reveal an increase in the vibrational relaxation lifetime of both amide I and carboxylate peak. Together with the enhanced spectral heterogeneity of the carboxylate band in the presence of CaCl_2_, these findings are indicative Ca^2+^ interaction at the amide CO and the carboxylate.

Ab initio MD simulations reveal that Ca^2+^ interacts with both of 2Alas binding sites, the amide CO group and the carboxylate group, but with a higher propensity at the negatively charged carboxylate. Computed VDOS align with the experimentally observed spectral shifts of both modes. The blueshift of the carboxylate ② with increasing CaCl_2_ concentration can be assigned to monodentate binding of not only one, but also two Ca^2+^ ions to the carboxylate, while a direct Ca^2+^- amide CO interaction only leads to minor spectral shifts.

The spectral response of the amide contrasts what has previously been observed for NMA, where interaction with Ca^2+^ leads to the complete dehydration of the amide CO, which was initially hydrated by two water molecules.^17^ Here, for 2Ala, the amide CO group is initially hydrated by (on average) less than one water molecule and the direct interaction with Ca^2+^ replaces one water molecule only. These differences in dehydration, together with vibrational Stark effects, can explain the differing spectral sensitivities to interaction with Ca^2+^.

The computational spectra and experiments demonstrate the necessity to consider all potential interaction sites within a peptide and competing binding to these sites in order to fully rationalize specific ion effects on peptides using vibrational signatures. Furthermore, our results highlight that the accessibility and hydration of both the peptide’s interaction sites and ions critically determine the sensitivity of amide modes to interaction with ions.

## Supporting Information Available

Synthesis of isotope-labeled 2Ala, difference linear IR spectra, linear IR spectra at different 2Ala concentrations and pH values, background subtraction and normalization of linear IR spectra, linear and 2D IR 2Ala-NaCl spectra, and analysis of CLS and vibrational relaxation lifetime, Gaussian fits of IR spectra, 2D IR spectra at further CaCl_2_ concentrations, detailed CLS and vibrational relaxation lifetime analysis for 2Ala-CaCl_2_ solutions, RDFs of 2Ala-water interaction, details on categorization for VDOS, DFT optimized geometries of 2Ala, energy level diagram of carboxylate mode, CO distances for bridging vs unbound carboxylate mode (PDF)
