# Supplementary material for: Deciphering Spectroscopic Signatures of Competing Ca2+ - Peptide Interactions
Source: J Phys Chem B. 2024 Oct 22;128(43):10688–98. doi: 10.1021/acs.jpcb.4c04760 (PMC11533179; doi:10.1021/acs.jpcb.4c04760)
Supplement: Supplementary file 1 — jp4c04760_si_001.pdf [file jp4c04760_si_001.pdf]

Supplementary Information

for

Deciphering Spectroscopic Signatures of Competing Ca^{2+} - Peptide Interactions

Carola S. Krevert,[†,¶] Lucas Gunkel,[†,¶] Johannes Sutter,[†] Raphael Meyer,[‡] Paul Schneider,[†] Yuki Nagata,[†] and Johannes Hunger[*,†]

†*Max Planck Insitute for Polymer Research, Department of Molecular Spectroscopy, Ackermannweg 10, 55128 Mainz, Germany*

‡*Max Planck Insitute for Polymer Research, Department of the Synthesis of Macromolecule, Ackermannweg 10, 55128 Mainz, Germany*

¶*Authors contributed equally*

E-mail: hunger@mpip-mainz.mpg.de

Synthesis of isotope-labeled 2Ala

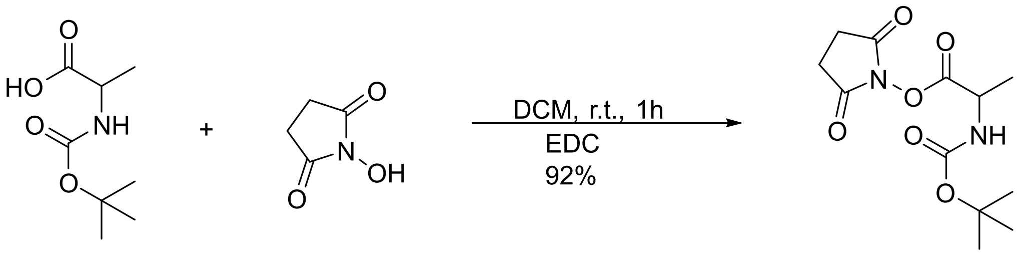

Figure S1: Step one in the reaction pathway for isotope-labeled 2Ala.

Boc-alanine (500 mg, 2.64 mmol), N-hydroxysuccinimide (304 mg, 2.64 mmol) and 1-ethyl-3-(3-dimethylaminopropyl)carbodiimide (EDC) (507 mg, 2.64 mmol) were dissolved in dichloromethane (DCM) (15 mL) and stirred at room temperature for 1 hour. The solution was washed with saturated $NaHCO_3$-solution and the aqueous phase extracted with DCM. The combined organic phases were washed with brine and the solvent removed under reduced pressure. The product was gained as a slightly rose solid without further purification (692 mg, 2.43 mmol, 92%).

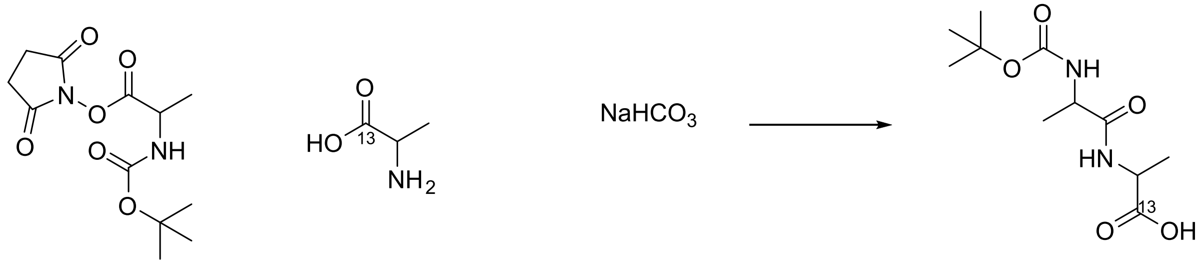

Figure S2: Step two in the reaction pathway for isotope-labeled 2Ala.

^{13}C-labeled alanine (43 mg, 0.48 mmol) and sodium bicarbonate (80 mg, 0.95 mmol) were dissolved in 10 mL tetrahydrofuran (THF)/H_2O (1:1). Boc-Ala-OSu (150 mg, 0.52 mmol) in 5 mL THF was added drop-wise to that mixture and stirred overnight. THF was removed under reduced pressure and the solution was acidified to pH 2 with 1 M HCl and subsequently extracted with ethyl acetate. The product was gained as a colorless solid (120 mg, 0.46 mmol)

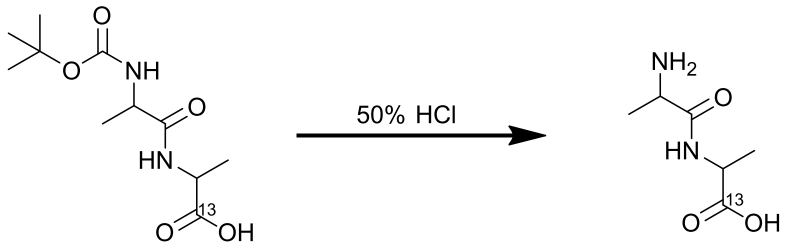

Figure S3: Step three in the reaction pathway for isotope-labeled 2Ala.

Boc-Ala-Ala (24 mg, 92 µmol) was suspended in 10 ml semiconcentrated HCl and stirred overnight. The solvent was then removed under reduced pressure to yield the product as a colorless solid (14 mg, 87 µmol). Before the measurement, we neutralized the acidic ^{13}C 2Ala with NaOH.

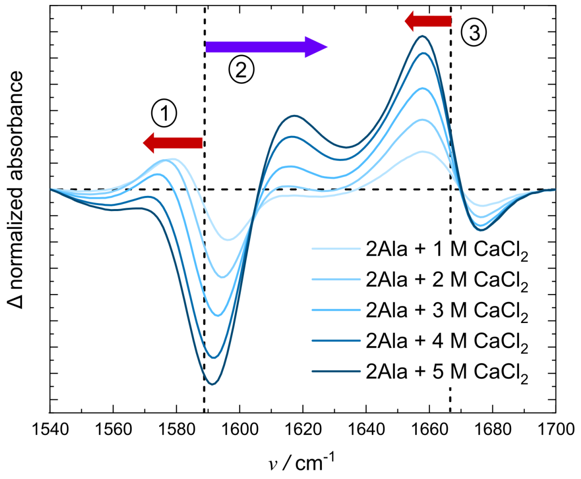

Figure S4: Difference spectra with the spectrum of 2Ala in D_2O subtracted from the 2Ala - $CaCl_2$ solutions at different salt concentrations to illustrate the three major trends with increasing salt concentration: At intermediate salt concentrations (1-3 M), a slight redshift can be observed for the carboxylate peak (①), while at higher salt concentrations (3-5 M) a significant blueshift is observed (②). The amide I mode redshifts as salt concentration is increased (③). The vertical dashed lines mark the peak positions of amide I and carboxylate in pure 2Ala solutions. Note that those difference spectra were obtained by subtracting area normalized spectra.

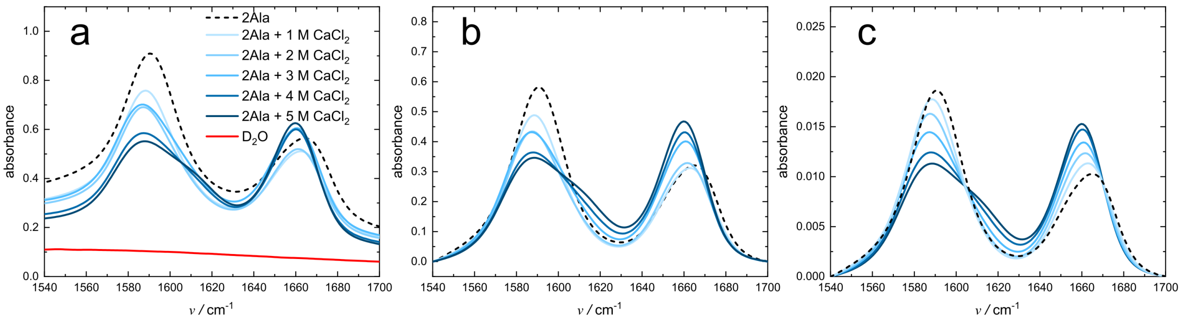

Figure S5: a) IR absorption spectra of 2Ala and 2Ala - $CaCl_2$ solutions as measured; b) linear background-subtracted spectra; c) linear background and area normalized spectra.

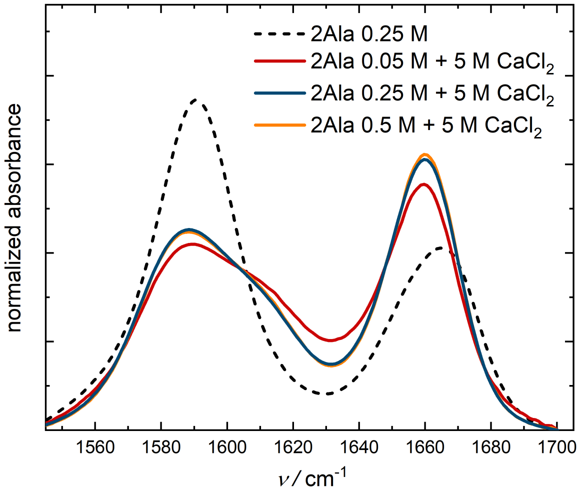

Figure S6: IR absorption spectra of 2Ala and 2Ala + 5 M $CaCl_2$ solution at 0.25 M 2Ala (dashed black line and solid blue line, respectively), as well as 0.05 M 2Ala, and 0.5 M 2Ala + 5 M $CaCl_2$ (solid red and orange lines). All spectra were background-subtracted and area-normalized. The spectra do not change significantly with altered 2Ala concentration even though the ratio peptide:salt changes significantly: At a wide range of 2Ala concentrations, the nature of interaction between peptide and ions is comparable.

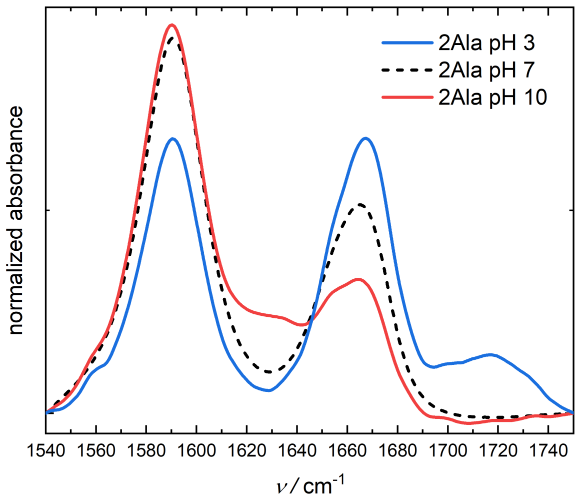

Figure S7: IR absorption spectra of 2Ala in D_2O at various pH values: pH 7 (black solid line), pH 3 (solid blue line) and pH 10 (solid red line). With increasing pH value, the NH_3^+ group is deprotonated, which changes the transition dipole moment of the amide I group significantly. A decrease in pH value results in a protonation of the carboxylate group, resulting in a decrease in signal of the asymmetric stretching vibration and the appearance of a mode due to the COOH group at 1710 cm^{-1}.

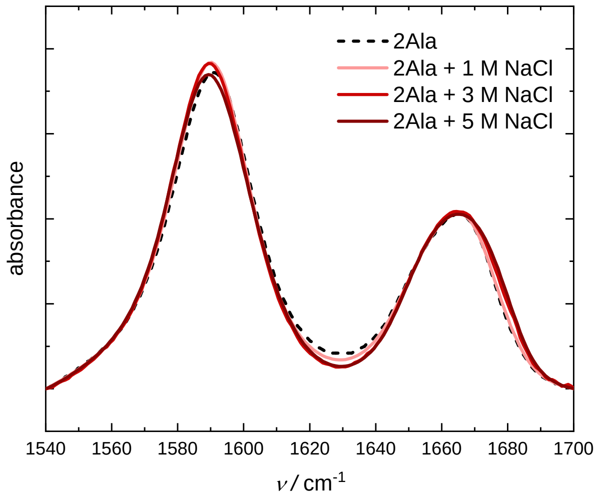

Figure S8: Linear, background subtracted and area-normalized IR spectra of 0.25 M 2Ala (black), 0.25 M 2Ala + 1 M NaCl (light red), 0.25 M 2Ala + 3 M NaCl (red), and 0.25 M 2Ala + 5 M NaCl (dark red).

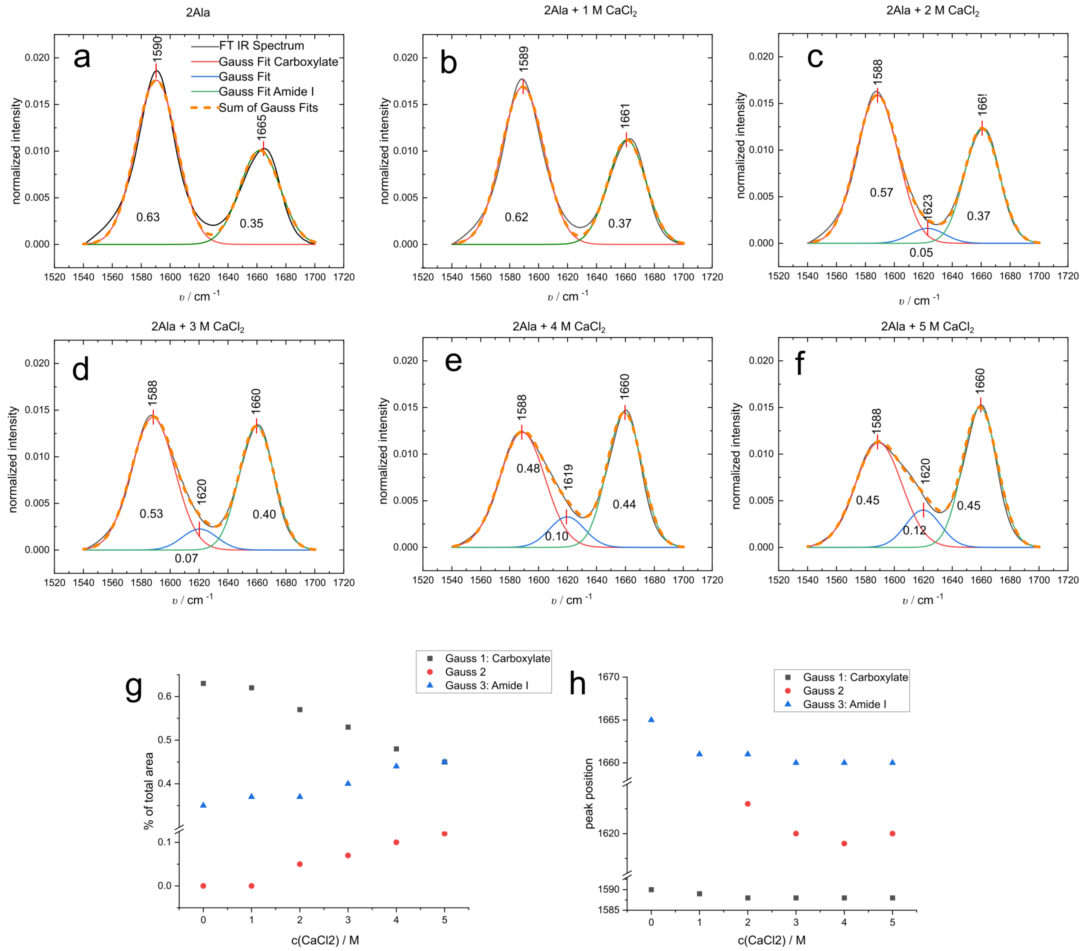

Figure S9: Linear IR spectra of 2Ala and 2Ala - $CaCl_2$ solutions with Gaussian fits (a-f). 0 M and 1 M $CaCl_2$ were fitted with two Gaussian functions, while 2-5 M $CaCl_2$ were fitted with a sum of three Gaussian functions. The concentration-dependent relative amplitudes (g) and peak positions (h) suggest that a third peak emerges with increasing $CaCl_2$ concentration.

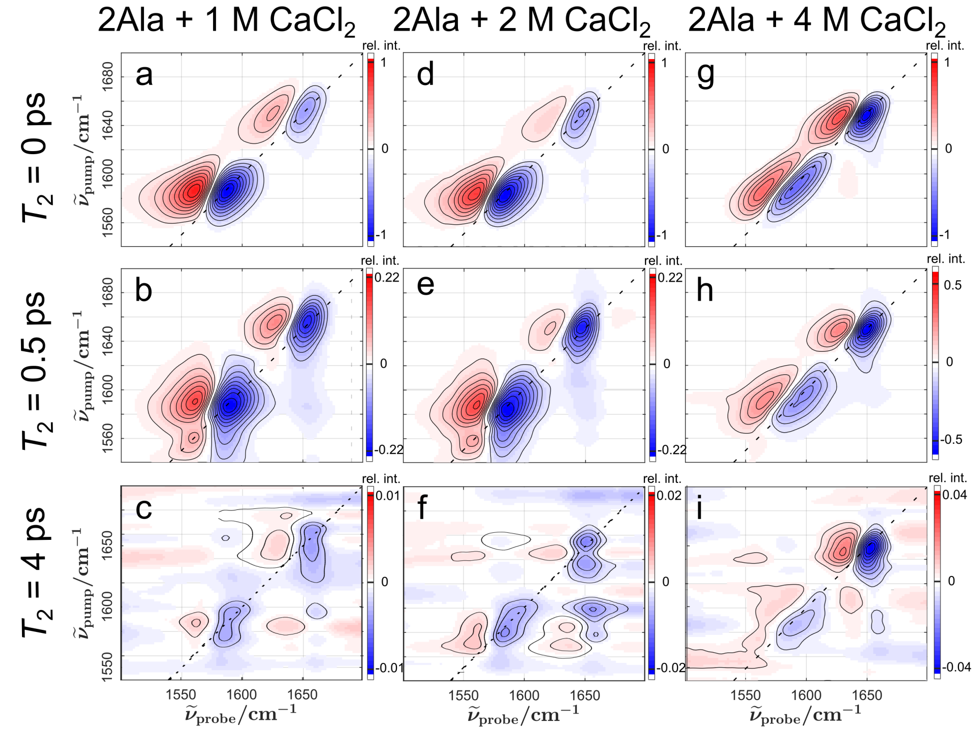

Figure S10: 2D IR spectra of 2Ala - $CaCl_2$ solutions at 1 M (a, b, c), 2 M (d, e, f), and 4 M (g, h i) $CaCl_2$ concentrations at 0 ps (a, d, g), 0.5 ps (b, e, h), and 4 ps (c, f, i) waiting time T_2. The observed trends resemble those shown in Figure 3 of the main manuscript.

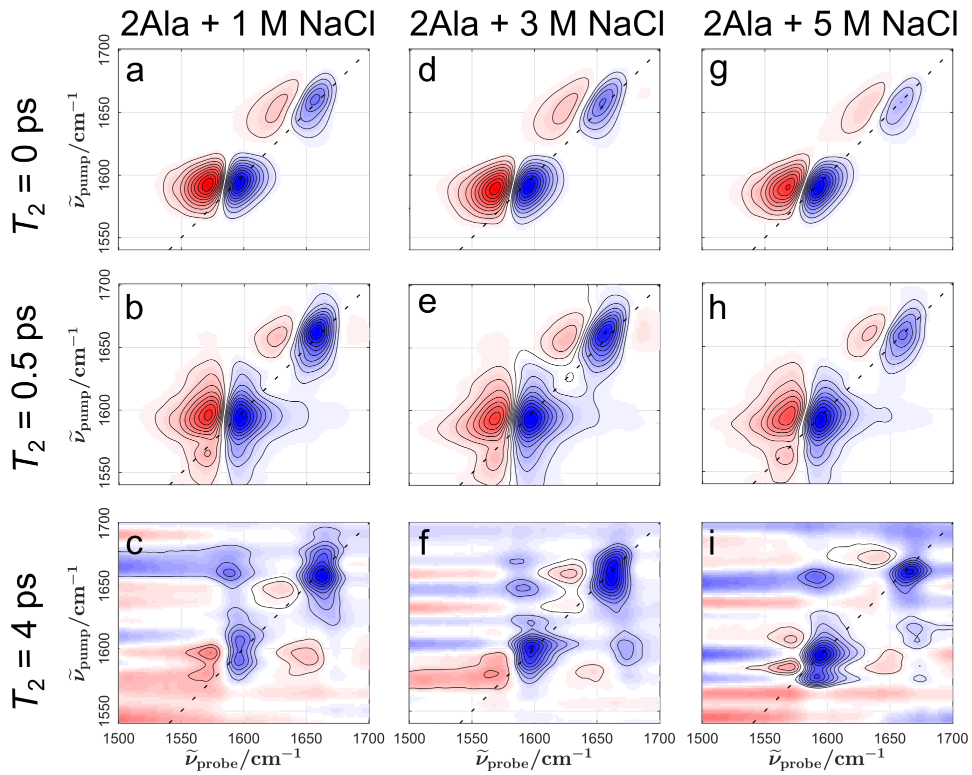

Figure S11: 2D IR spectra of aqueous 2Ala NaCl solutions at 1, 3 and 5 M NaCl concentration and 0 ps, 0.5 ps and 4 ps waiting time. The 2D spectra resemble the trends in the linear IR spectra of aqueous 2Ala-NaCl solutions: Only very moderate NaCl-induced changes in peak shape or vibrational dynamics can be observed.

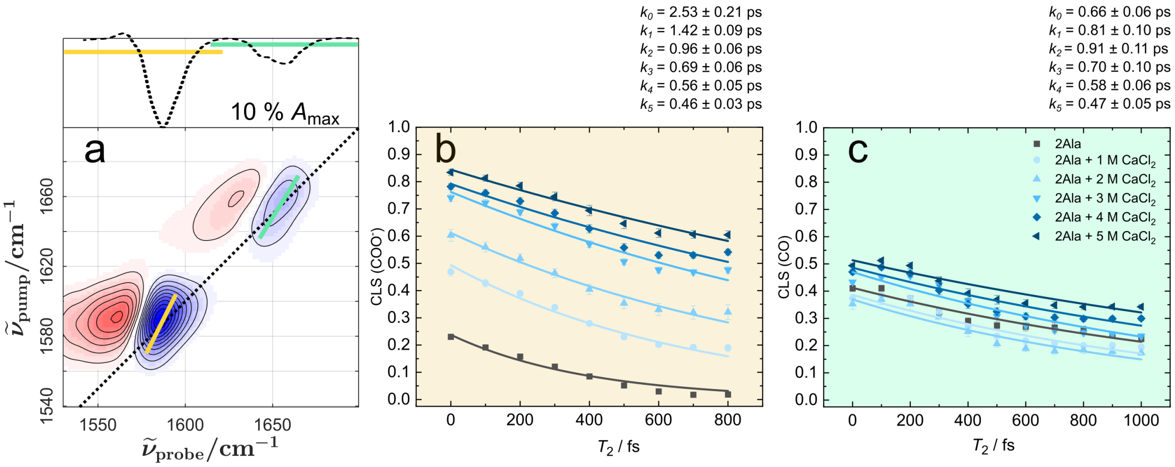

Figure S12: a) 2D IR spectrum of 2Ala at 0 fs waiting time with diagonal cut shown in the top panel. CLS were evaluated with the center line positions at frequencies for which the signal was less than 10 % of the bleach maximum (see yellow and cyan lines). The frequency range over which the CLS was evaluated was kept constant at all waiting times. The concentration- and waiting-time-dependent values for the CLS are displayed in b for amide I and c for carboxylate. The CLS decays were modelled with an exponential function ($CLS(T_2) = CLS(T_2 = 0) \cdot \exp(-k_{\mathrm{CLS}} \cdot T_2)$.

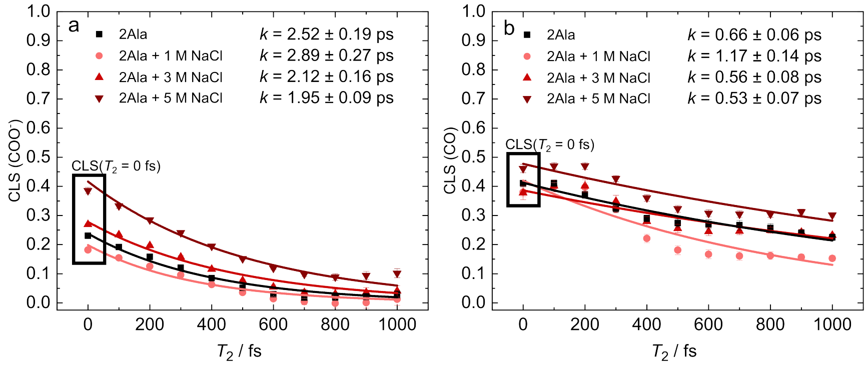

Figure S13: Waiting-time-dependent values for CLS of amide I (CO) and carboxylate mode (COO^-) in the presence of NaCl. Solid lines show the exponential fit (compare Figure S12). The respective values for the decay rates k are indicated in the Figure. The rate constant decreases by about 20 % for the case of the COO^- mode, while the rate constant of the amide I mode (CO) decreases by about 15 %.

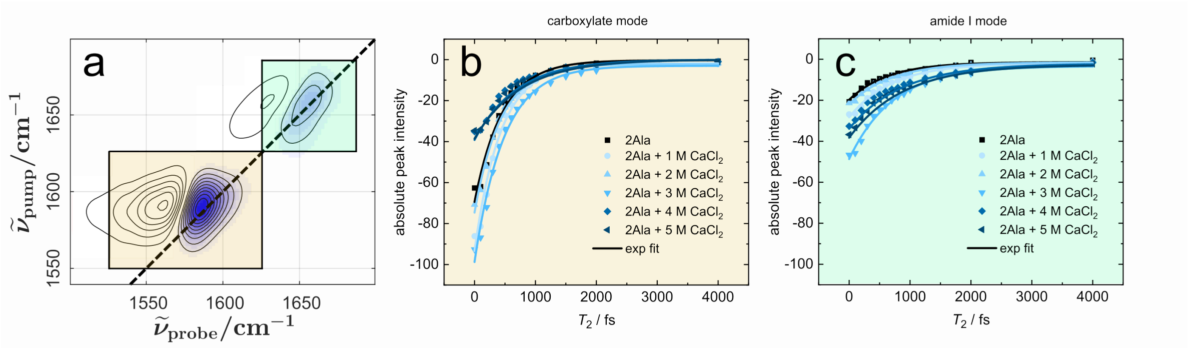

Figure S14: Vibrational relaxation lifetime determined via area integration of all negative values in the marked area (a) of both the carboxylate mode (b) and the amide I mode (c) and fitting them exponentially with Equation 1 (main manuscript). The vibrational energy relaxation times increase significantly with increasing concentration of $CaCl_2$ (Figure 4b).

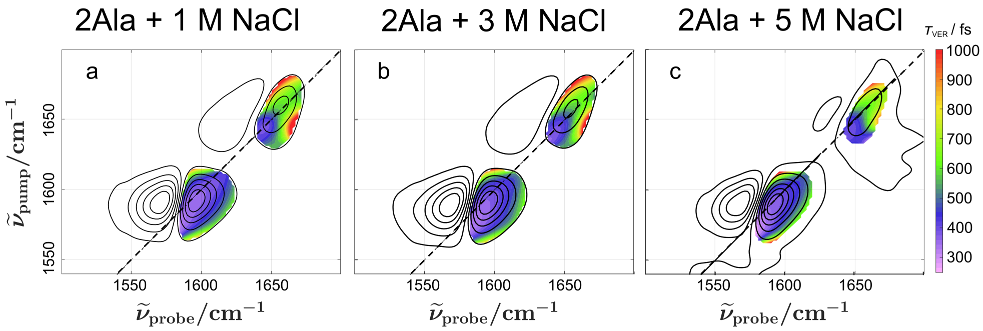

Figure S15: Decay time maps for 2Ala-NaCl solutions. The decay times determined for the entire peak (see Figure S14) gives relaxation lifetimes of: 1 M: amide I: 630 ± 40 fs; carboxylate: 480 ± 60 fs; 3 M: amide I: 610 ± 40 fs; carboxylate: 490 ± 60 fs; 5 M: amide I: 600 ± 40 fs; carboxylate: 510 ± 60 fs. Salt induced changes to the decay times are less pronounced for 2Ala-NaCl solutions, as compared to 2Ala-$CaCl_2$.

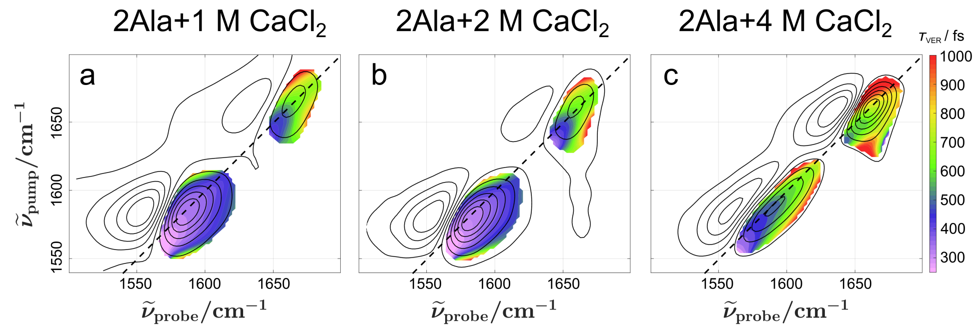

Figure S16: Decay time maps for 2Ala-$CaCl_2$ solutions for 1 M (a), 2 M (b) and 4 M $CaCl_2$ (c) solution (see also Figure 5 of the main manuscript).

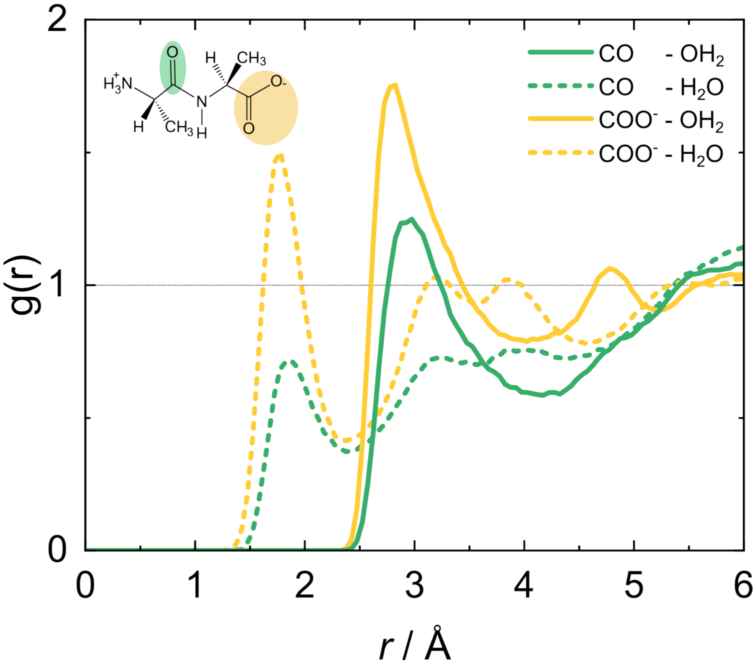

Figure S17: Radial distribution function of 2Ala carboxylate oxygen (yellow) and amide oxygen (green) with water hydrogen (dashed) and oxygen (solid) in the presence of 5 M $CaCl_2$.

Categorization

For the calculation of the VDOS of different binding configurations we categorize simulation frames based on the interaction of the three oxygen atoms of 2Ala (COO1, COO2 and CO) with Ca^{2+}. We take the first minimum in the CO-Ca RDF (see Figure 6a) at 3.1 Å as a maximum value for being classified as direct interaction. Additionally, for a frame to count towards a certain category the Ca-CO distance has to be smaller than the threshold at this frame and a frame 1 ps later. We then check the occupation of the three binding sites and categorize frames accordingly. These categories together with their occurrence are listed below in Table S1. We display VDOS spectra for categories 1-3 in Figure 6b and for categories 4 and 5 in Figure 6c. We see no evidence for all interaction sites being occupied at the same time (category 6). Note that 1% of the frames are categorized as transitional frames where calcium -2Ala interactions are formed/broken.

Table S1: Categories for calculation of VDOS and their occurrence.

Category	number of Ca^{2+} at carboxylate	number of Ca^{2+} at amide CO	occurrence (%)
1	0	0	32
2	1	0	26
3	2	0	26
4	0	1	2
5	1	1	13
6	2	1	0

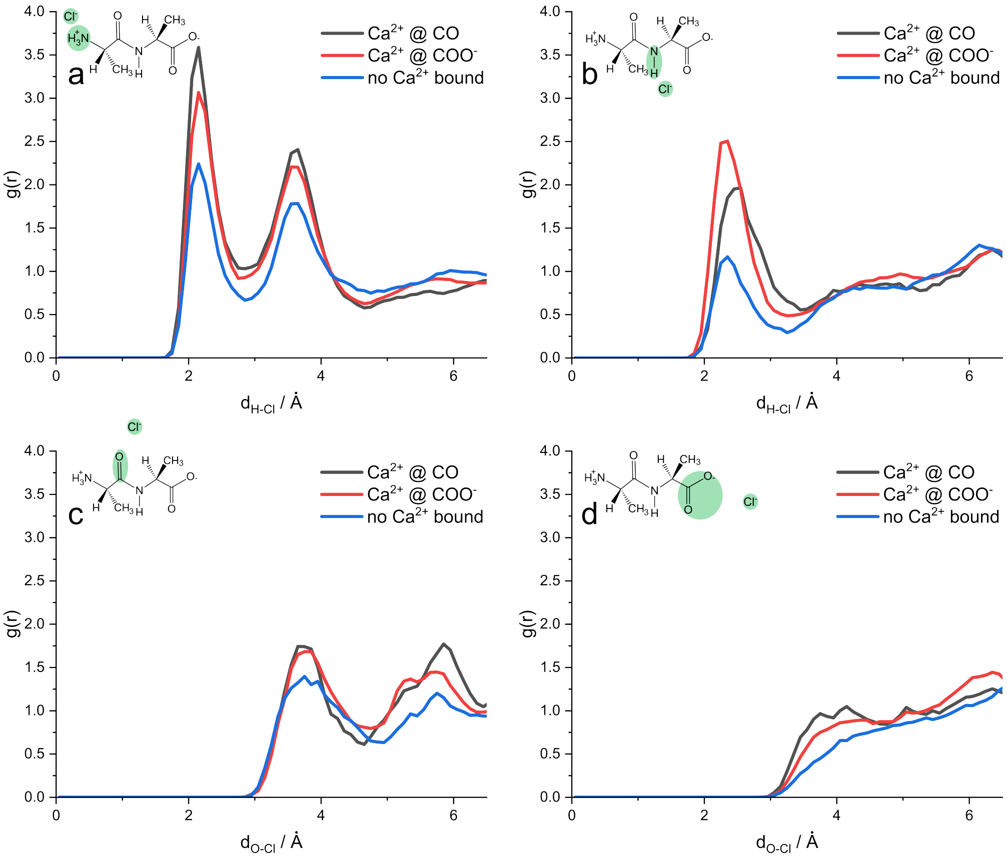

Figure S18: H-Cl^- radial distribution functions for a) the N-terminus and b) the amide NH group. Panels c) and d) show the O-Cl^- radial distribution functions for the amide CO and the C-terminus, respectively. To explore correlations of Cl^- interaction to Ca^{2+} binding, we show RDFs separately for Ca^{2+} bound to the amide CO (black line), Ca^{2+} bound to the carboxylate (red line), and no Ca^{2+} bound to 2Ala (blue line). Cl^- directly interacts with all NH protons and the occupancy increases when Ca^{2+} is bound to 2Ala, while no Cl^- ions are in direct contact with 2Alas oxygens.

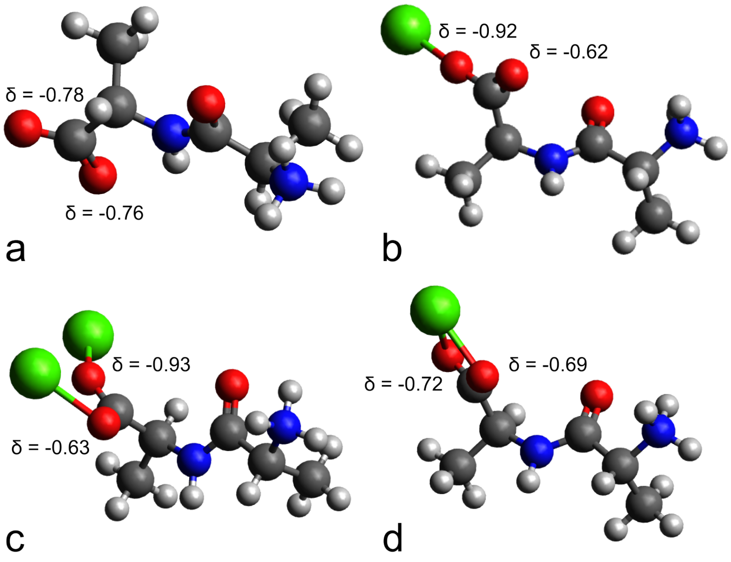

Figure S19: DFT optimized geometries of 2Ala with a) 0 Ca^{2+}, b) 1 Ca^{2+} monodentate, c) 2 Ca^{2+} monodentate, and d) 1 Ca^{2+} bidentate geometries together with calculated partial charges for carboxylate oxygens to illustrate the degeneracy of both CO groups.

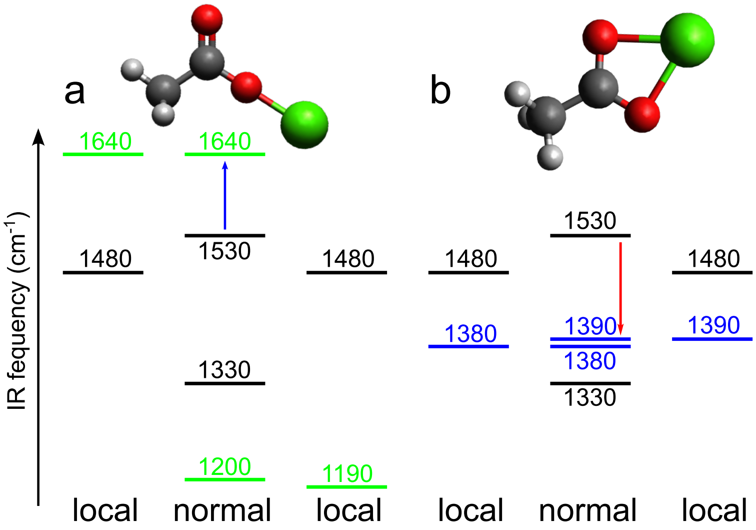

Figure S20: Energy level diagram for the carboxylate modes with a) pseudo bridging and b) bidentate binding of 1 Ca^{2+} to an acetate anion as obtained from single point DFT calculations. The harmonic, coupled normal modes are shown in the center for the acetate (black) and the acetate with Ca^{2+} (green in panel a) and blue in panel b)). Left and right energy levels correspond to the local (decoupled) CO stretching mode frequencies, which we obtained by artificially increasing the mass of one of the oxygens and the atoms of the methyl group to 100 u. Only for acetate in the absence of Ca^{2+} coupling of the two local CO modes results in an appreciable splitting into symmetric and anti-symmetric stretching modes. This splitting is not symmetric, presumably due to C-H mode contributions.[1] In the presence of Ca^{2+}, coupling is weak and the coupled harmonic frequencies resemble the local mode frequencies, demonstrating the lifting of the degeneracy.

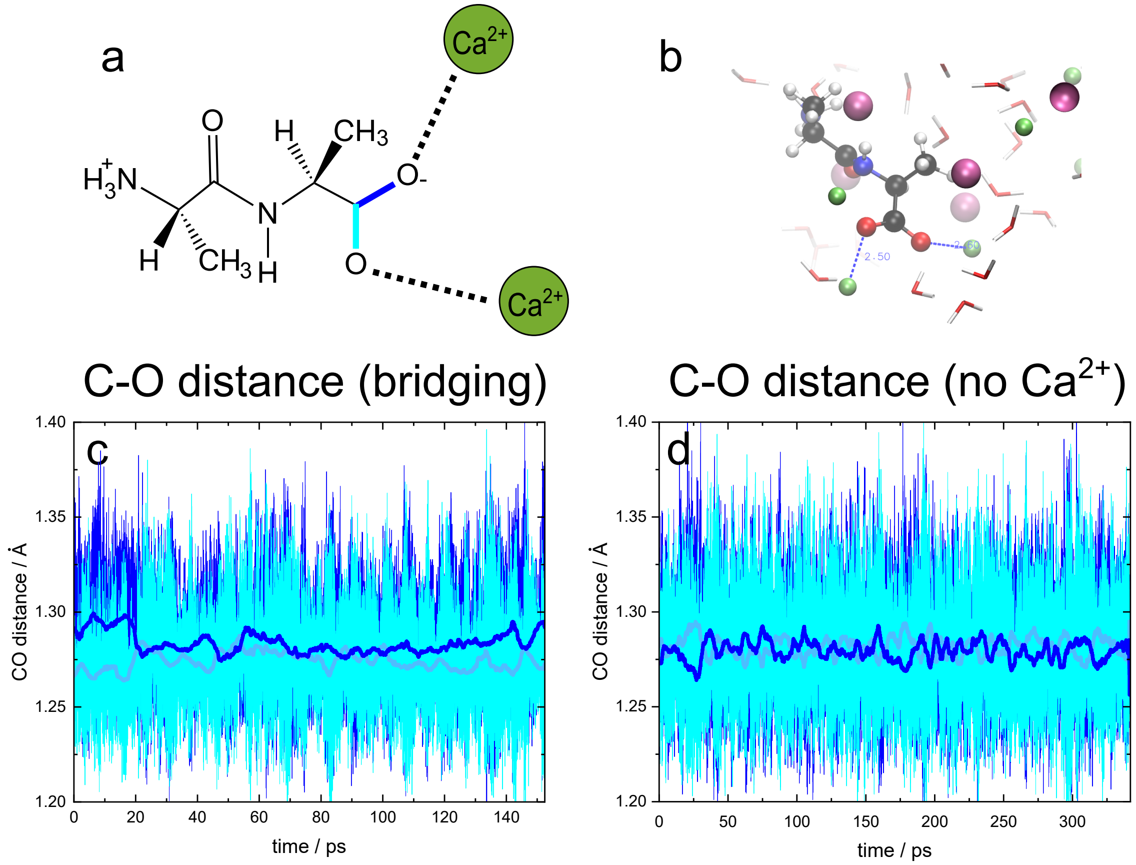

Figure S21: a) Schematic drawing of 2Ala interacting in a bridging configuration with the CO bonds color-coded. b) Simulation snapshot of 2Ala in a bridging geometry. CO distances versus time for a trajectory in bridging geometry c) and without direct Ca^{2+} interaction d). The colors of the solid lines in c) and d) represent the distances with the same color as in a). The solid lines mark the moving median distances. The mean difference between both CO distances of 0.01 $\mathring{A}$ for the bridging and of 0.002 $\mathring{A}$ in the absence of Ca^{2+} illustrates the absence of the degeneracy of both CO potentials for the bridging configuration.

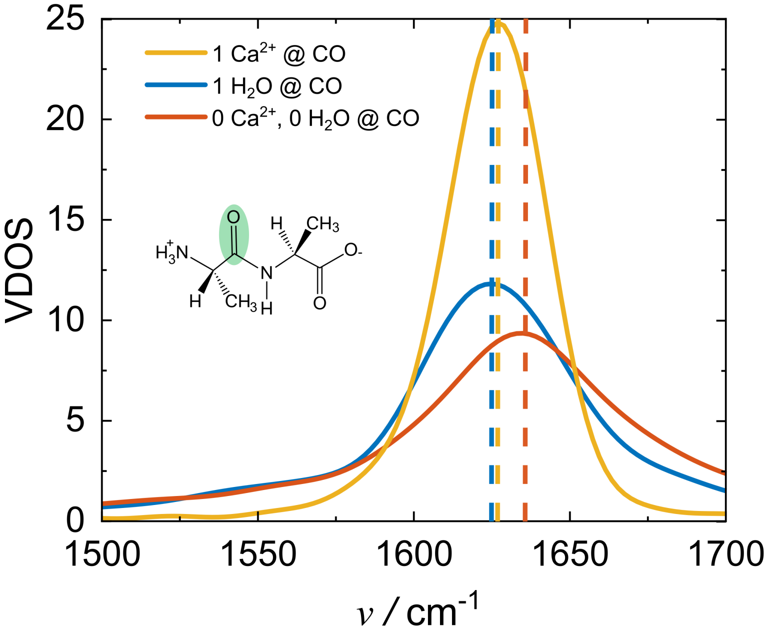

Figure S22: VDOS of the amide CO response for direct amide-Ca^{2+} interaction (yellow), direct amide-water interaction (blue), and no interaction of amide with water or Ca^{2+} (orange). Interaction with Ca^{2+} or water induces a redshift of similar magnitude.

References

(1) Mitra, S.; Werling, K.; Berquist, E. J.; Lambrecht, D. S.; Garrett-Roe, S. CH Mode Mixing Determines the Band Shape of the Carboxylate Symmetric Stretch in Apo-EDTA, Ca 2+ –EDTA, and Mg 2+ –EDTA. *The Journal of Physical Chemistry A* **2021**, *125*, 4867–4881.
